# Paralogs and mutants show that one DMA synthase functions in iron homeostasis in rice

**DOI:** 10.1093/jxb/erx065

**Published:** 2017-03-28

**Authors:** Khurram Bashir, Tomoko Nozoye, Seiji Nagasaka, Sultana Rasheed, Nanako Miyauchi, Motoaki Seki, Hiromi Nakanishi, Naoko K. Nishizawa

**Affiliations:** 1 Graduate School of Agricultural and Life Sciences, The University of Tokyo, 1-1-1 Yayoi, Bunkyo-ku, Tokyo 113–8657, Japan; 2 Center for Sustainable Resource Science, RIKEN Yokohama Campus, 1-7-22 Suehiro-cho, Tsurumi-ku, Yokohama City, Kanagawa, 230-0045, Japan; 3 Center for Liberal Arts, Meiji Gakuin University, 1518 Kamikurata-cho, Totsuka-ku, Yokohama 244–8539, Japan; 4 Graduate School of Life Sciences, Toyo University, 1-1-1 Izumino Itakura-machi, Gunma 374-0193, Japan; 5 CREST, JST, 4-1-8 Honcho, Kawaguchi, Saitama 332-0012, Japan; 6 Research Institute for Bioresources and Biotechnology, Ishikawa Prefectural University, 1–308 Suematsu, Nonoichi-shi, Ishikawa 921–8836, Japan

**Keywords:** Aldo-keto reductase, deoxymugineic acid synthase, DMA, iron, iron deficiency, *Oryza sativa*, phytosiderophores, zinc.

## Abstract

Rice (*Oryza sativa*) secretes 2′-deoxymugineic acid (DMA) to acquire insoluble iron (Fe) from the rhizosphere. In rice, DMA is synthesized by DMA synthase 1 (OsDMAS1), a member of the aldo-keto reductase super family. We screened OsDMAS1 paralogs for DMA synthesis. None of these paralogs displayed *in vitro* DMA synthesis activity, suggesting that rice only harbors one functional DMAS. We further characterized *OsDMAS1* mutant plants. We failed to screen homozygous knock-out plants (*dmas-1*), so we characterized DMAS knock-down plants (*dmas-kd1* and *dmas-kd2*). Under Fe-deficient conditions, *dmas-kd1* plants were more chlorotic compared to the wild-type (WT) plants, and the expression of *OsNAS3*, *OsYSL2*, *OsIRT1*, and *OsIRO2* was significantly up-regulated in the *dmas-kd1* mutant, indicating that metal homeostasis was significantly disturbed. The secretion of DMA in *dmas-kd1* was not significantly reduced. The *dmas-kd1* plants accumulated less Fe in their roots compared to WT plants when grown with 10 μM FeSO_4_. The *dmas-kd1* plants accumulated more Zn in their roots compared to WT plants under Fe-deficient, Fe-EDTA, and FeSO_4_ conditions. In both dehusked rice seeds (brown rice) and polished rice, no differences were observed for Fe, Cu, or Mn accumulation, whereas *dmas-kd1* seeds significantly accumulated more Zn in brown rice. Our data suggests that rice only harbors one functional gene for DMA synthesis. In addition, the knock-down of *OsDMAS1* significantly up-regulates the genes involved in Fe uptake and homeostasis.

## Highlight

Rice only harbors one functional gene for DMA synthesis, and the knock-down of *OsDMAS1* significantly up-regulates the genes involved in Fe uptake and homeostasis.

## Introduction

Iron (Fe) and zinc (Zn) are essential micronutrients for living organisms, and deficiency of these minerals can lead to serious nutritional problems (World Health Organization, 2002; Lingam *et al.*, 2011; Bashir *et al.*, 2013*c*). Plants depend on Fe for several essential cellular processes, such as respiration, chlorophyll biosynthesis, and photosynthetic electron transport; thus, it is not surprising that plant growth and metabolism is significantly impaired under Fe-deficient conditions (Guerinot and Yi, 1994; Bashir *et al.*, 2016; Vigani *et al.*, 2016). Despite the fact that mineral soils contain more than 5% Fe, its availability under neutral pH conditions is estimated to be around 10^−17^ M_,_ whereas plants require Fe in the range of 10^−9^–10^−4^ M (Guerinot and Yi, 1994; Guerinot, 2010). Fe exists largely as sparingly soluble ferric complexes under aerobic conditions. Thus, in soils with a high pH (calcareous soils), the absorption of Fe and other metals (e.g. Zn and Mn) is particularly problematic (Ishimaru *et al.*, 2011*b*; Bashir *et al.*, 2012). Plants have evolved sophisticated mechanisms in order to take up Fe from the soil, termed Strategy I and Strategy II mechanisms (Marschner *et al.*, 1986; Mori, 1999). Strategy I plants secrete protons to lower the rhizosphere pH, reduce Fe(III) to a more soluble ferrous form at the root surface by ferric-chelate reductase (FRO), and transport ferrous ions by IRT1 (Eide *et al.*, 1996; Vert *et al.*, 2002). Strategy I plants also secrete phenolic compounds such as coumarins to acquire Fe (Jin *et al.*, 2007; Schmid *et al.*, 2014; Clemens and Weber, 2016; Sisó-Terraza *et al.*, 2016). Rice (*Oryza sativa*) is reported to adopt a partial Strategy I, using the secretion of phenolics and OsIRT1, but not FRO, to take up Fe^2+^, which seems to have evolved under submerged paddy fields (Ishimaru *et al.*, 2006; Bashir *et al.*, 2011*a*).

Strategy II plants (which are graminaceous monocots), synthesize and secrete mugineic acid family phytosiderophores (MAs) from their roots via transporter of mugineic acid 1 (TOM1) to solubilize Fe (Takagi, 1976; Takagi *et al.*, 1984; Nozoye *et al.*, 2011). The resulting Fe-MA complexes are then reabsorbed into the roots via the yellow stripe 1 (YS1) family transporters (Curie *et al.*, 2001; Inoue *et al.*, 2009; Lee *et al.*, 2009*a*; Nozoye *et al.*, 2011). The biosynthetic pathway for MAs has been characterized in detail. Synthesis of MAs starts with the trimerization of S-adenosylmethionine (SAM) (Mori and Nishizawa, 1987; Shojima *et al.*, 1989, 1990) into nicotianamine (NA) by NA synthase (NAS) (Higuchi *et al.*, 1999). NA serves as a ubiquitous metal chelator in all plants, as well as a substrate that is converted into a 3′′-keto intermediate by NA amino transferase (NAAT) (Takahashi *et al.*, 1999). Subsequent reduction of the 3′′-carbon of the keto intermediate by deoxymugineic acid synthase (DMAS) (Bashir *et al.*, 2006) produces deoxymugineic acid (DMA). DMA is then released into the rhizosphere via the TOM1 transporter (Nozoye *et al.*, 2011), where it binds to insoluble Fe, and the Fe-DMA complex is taken up by OsYSL15 (Inoue *et al.*, 2009). Recently, the contribution of TOM2 to normal plant growth through regulating the internal transport of DMA has also been documented (Nozoye *et al.*, 2015). The genes involved in MA biosynthesis have been characterized in rice, and most are up-regulated by Fe deficiency, resulting in increased secretion of MAs (Bashir et al., 2013*a*, 2014; Kobayashi *et al.*, 2014). In roots the expression of *OsDMAS1* changes with Fe availability, and not only increases under Fe deficiency but also decreases under excess Fe conditions (Bashir *et al.*, 2014). Tolerance to Fe-deficiency stress is strongly correlated with the MAs secreted in graminaceous plants. Rice synthesizes and secretes DMA in relatively low amounts, which makes it sensitive to Fe deficiency when grown in aerobic soil conditions (Mori *et al.*, 1991; Mori, 1999). Three enzymes, OsNAS1–3, are responsible for the trimerization of SAM. OsNAAT1 converts NA to a 3′′-keto intermediate, which is further converted into DMA by OsDMAS1 (Inoue et al., 2003, 2008; Bashir *et al.*, 2006). In addition to the differences between Strategy I and II plants in the uptake of Fe from the rhizosphere, Strategy II plants have the advantage of metal homeostasis within the plant body due to the presence of MAs in addition to other Fe chelators, such as NA, citrate, and phenolics (Ishimaru *et al.*, 2011*c*; Bashir *et al.*, 2011*a*, 2013*a*).

After their acquisition from the soil, metals are transported to leaves and developing seeds through a series of complex molecular mechanisms (Bashir et al., 2011a;Ishimaru *et al.*, 2011*c*; Yamaji and Ma, 2014). Any disturbance in cellular Fe homeostasis significantly alters cellular metabolism (Kim *et al.*, 2006; Bashir *et al.*, 2011*b*, 2013*b*;Vigani *et al.*, 2016). The YSL family of oligopeptide transporters plays a significant role in the distribution of Fe to shoots and inflorescences. OsYSL2 transports Fe(II)-NA and Mn(II)-NA, and is important for the translocation of Fe and Mn via the phloem (Koike *et al.*, 2004; Ishimaru *et al.*, 2010), whereas OsYSL16 plays a role in the allocation of Fe(III)-DMA via the vascular bundles (Kakei *et al.*, 2012).

The role of DMA in metal homeostasis has been studied extensively. DMA has been detected in root exudates, roots, shoots, and seeds as well as in the xylem and phloem of rice, and is believed to play a very significant role in Fe and metal transport in rice (Kakei *et al.*, 2009; Nishiyama *et al.*, 2012; Ariga *et al.*, 2014; Nozoye *et al.*, 2014); however, mutants for DMAS genes have not been characterized. Moreover, the existence of multiple genes for *NAS* and *NAAT* in graminaceous crops (Inoue *et al.*, 2003, 2008) indicates the possibility that DMAS may have functional paralogs in rice and other monocots. In this study, we cloned selected paralogs of *OsDMAS1* from rice and tested whether these paralogs could convert the 3′′-keto intermediate to DMA *in vitro*. The characterization of OsDMAS1 paralogs and mutants significantly advances our understanding of metal uptake and translocation in rice. Changes in the metal profiling and expression of metal homeostasis-related genes highlight the role of *OsDMAS1* in Fe homeostasis in rice.

## Materials and methods

### Cloning of *OsDMAS1* paralogs and enzyme assay

Paralogs of *OsDMAS1* belonging to the aldo-keto reductase family were identified using BLAST (http://www.ncbi.nlm.nih.gov/BLAST/). The predicted full-length amino acid sequences were used to generate an unrooted phylogenetic tree with ClustalW using DNASIS software (Hitachi Software). Four paralogs (AK102609, AK068616, AK067910, and AK103553) were selected to check whether these paralogs possessed DMAS activity. Selected paralogs were cloned into pENTR/D-TOPO (Invitrogen, Carlsbad, CA, USA) and sequenced using an ABI sequencer (Applied Biosystems). These genes were then subcloned into pMAL-c2, and respective fusion plasmids were introduced into the *Escherichia coli* strain XL1-Blue to produce maltose-binding protein (MBP) fusion proteins. The cells were induced to produce recombinant MBP fusion proteins, and these were then purified using amylose resin, as described previously (Higuchi *et al.*, 1999). The size of the purified proteins was confirmed by SDS-PAGE. The amino acid sequences of graminaceous DMAS proteins and rice paralogs of OsDMAS1 were aligned, and the NADPH-binding domain and the putative DMAS active site were recognized as previously described (Jez *et al.*, 1997; Mindnich and Penning, 2009).

DMAS activity was determined exactly as described previously (Bashir *et al.*, 2006). Briefly, 5 μg HvNAAT-A fusion protein/reaction was centrifuged in an Amicon Ultrafree-MC 30-kDa-cut-off filter unit (Millipore) at 6200 *g* at 4 °C for 15 min. The flow-through was discarded, and 50 μl TAPS buffer (50 mM TAPS, 5 mM KCl, 5 mM MgCl_2_, 10 mM 2-oxoglutaric acid, 10 μM pyridoxal-5′-phosphate, and 150 μM nicotianamine) was added, mixed by pipetting, and incubated at 26 °C for 30 min. The filter was then placed in a new Eppendorf tube and centrifuged at 6200 *g* at 4 °C for 15 min. The flow-through was collected, and NADPH was added to a final concentration of 25 μM. The protein samples to be assayed (1 μg/reaction) were placed in new filter units and centrifuged at 6200 *g* at 4 °C for 1 min. All of the samples were prepared separately, including OsDMAS1 and HvDMAS1 as positive controls. Then, 46 μl of flow-through containing the 3″-keto intermediate and NADPH (prepared as described above) were added to each filter unit containing DMAS or its paralogs, mixed, and incubated at room temperature for 30 min. Samples were filtered, and 50 μl of each sample was analysed by HPLC using purified DMA as a standard.

### Characterizing *OsDMAS1* mutants

Three *OsDMAS1* T-DNA mutant lines *dmas-kd1*, *dmas-kd2*, and *dmas-1* were obtained from the RiceGE rice functional genomics database (http://signal.salk.edu/cgi-bin/RiceGE;Jeon *et al.*, 2000; Jeong *et al.*, 2006). Isolation of the *dmas-kd1* homozygous mutant was performed by PCR-based screening using a T-DNA right-border-specific primer, 5′-AATATCTGCATCGGCGAACTGATCG-3′, and the *OsDMAS1*-specific primers 5′-TTCAGTATCTC TTATCTACCCATCCA-3′ and 5′-TGATAATCCAAGTGG CGTTCT-3′. For *dmas-kd2 OsDMAS1*, the specific primers were 5′-AGAACGCCACTTGGATTATCA-3′ and 5′-CCCCTCTC TTGGACGGACTGCGG-3′. For *dmas-1*, the *OsDMAS1*-specific primers were 5′-GATGGAGTACGTGGACTTGTAC-3′ and 5′-ACAGCTCCCTCAGCTTCCTCT-3′. Rice seeds (*Oryza sativa* L. cv. dongjin) were germinated on wet filter paper and cultured under a 14-h photoperiod at 320 μmol photons m^−2^ s^−1^ at 25 °C in the light and 20 °C in the dark as described previously (Bashir *et al.*, 2007). For Fe-deficiency treatments, plants were transferred to a culture solution lacking Fe. Roots and leaves were harvested after 7 d, frozen in liquid nitrogen, and stored at –80 °C until use. RNA was extracted from the roots and shoots of four plants for each replication. To compare metal accumulation, 3-week-old wild-type (WT) and *dmas-kd1* plants were grown in hydroponic solution as described above without Fe, in the presence of 100 μM Fe-EDTA or 10 µM FeSO_4_ for 1 week. Replication was four plants for each treatment.

For identification of *dmas-1* homozygous seeds, DNA was extracted from individual seeds of WT and *dmas-1* (*n*=24) using QuickExtract™ seed DNA extraction solution according to the manufacturer’s protocol (Epicentre, USA). The presence of T-DNA and the homo/heterozygous status of *dmas-1* were determined by PCR analysis using primers specific for *dmas-1* and T-DNA, as described above. Data were analysed as described previously (Bashir *et al.*, 2011*b*).

For measurement of secreted DMA, 2-week-old plants were subjected to Fe deficiency for 7 d. At this stage, roots were rinsed with deionized water and the exudates from WT and *dmas-kd1* plants were collected for six biological replicants for 4 h, from the start of the illumination period. The antimicrobial agent Micropur (Katadyn Products Inc., Wallisellen, Switzerland) was added to the water to prevent microbial degradation of DMA. The exudates were filtered through Advantec 5C filter paper (Toyo Roshi Kaisha Ltd, Tokyo, Japan). The cationic fraction of the root exudate was eluted by 2N NH_4_OH eluate from Amberlite IR(H^+^)120 (Rohm and Haas Co., Philadelphia, PA, USA) as described previously (Mori *et al.*, 1987; Suzuki *et al.*, 2006). Samples were condensed, microfiltered, and DMA secretion was quantified using HPLC as described previously (Mori *et al.*, 1987).

### Real-time PCR analyses

Samples were prepared as described above. Total RNA was extracted from the roots of *dmas-kd1* or WT plants using an RNeasy Plant Kit (Qiagen, Valencia, CA, USA) following the manufacturer’s instructions. Using ReverTra Ace qPCR RT Master Mix with genomic DNA Remover (Toyobo, Tokyo, Japan), contaminated genomic DNA was removed from the total RNA, and first-strand cDNA was synthesized. For RT analysis, the primers for *dmas-kd1* and *dmas-kd2* were forward 5′-GAGGAGGAGAGGCAGAGGAT-3′ and reverse 5′-TCAACACGATCGTCAAGAGC-3′. The primers used for the qPCR of *OsNAS1–3*, *OsNAAT1*, *OsYSL15*, *OsYSL2*, *OsIRT1*, and *OsIRO2* were as described previously by Kobayashi *et al.* (2009). The primers used for the qPCR of *TOM1* and *OsActin1* were as previously described by Nozoye *et al.* (2011). The fold-change between two samples was calculated according to the comparative CT method (Schmittgen and Livak, 2008) and expressed as copies/*OsActin 1*.

### Determination of metal concentrations

The elemental analysis of the WT and *dmas-kd1* knock-out plants was performed using inductively coupled plasma atomic emission spectroscopy (ICP-AES; SPS1200VR; Seiko, Tokyo, Japan), and expressed as µg g^–1^ dry weight as previously described (Ishimaru et al., 2009, 2011*a*; Bashir *et al.*, 2013*b*, 2014).

## Results

### OsDMAS1 paralogs do not exhibit DMAS activity

**Fig. 1.**
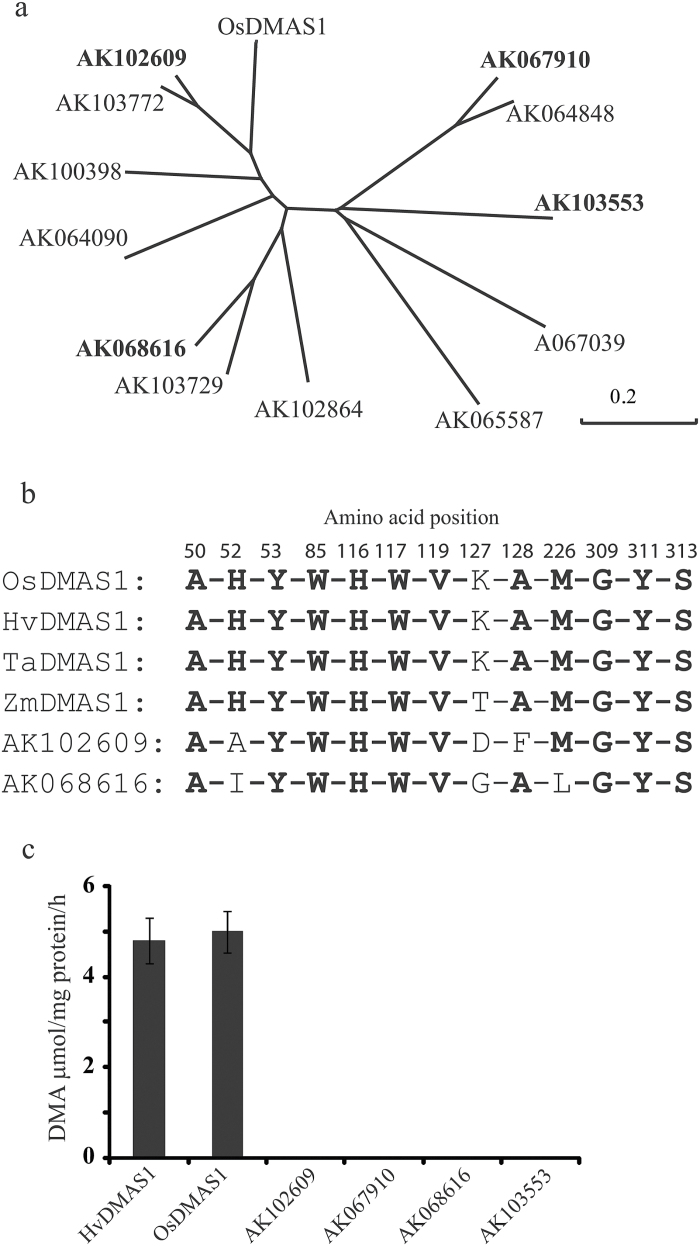
OsDMAS1 paralogs do not process *in vitro* DMAS activity. (a) Phylogeny of OsDMAS1 paralogs. (b) Substrate-binding site of graminaceous DMASs and selected paralogs of OsDMAS1. (c) *In vitro* DMAS enzyme activity of HvDMAS1, OsDMAS1, and four paralogs of OsDMAS1.

OsDMAS1 is a member of the aldo-keto reductase (AKR) superfamily. Members of this family are typically monomeric (α/β)_8_ barrel proteins, approximately 320 amino acids in length, which depend on NADPH to metabolize an array of substrates (Jez *et al.*, 1997). We searched for rice paralogs to check whether other members also catalyse 3’’-keto acid into DMA. We identified 13 paralogs in rice and cloned AK102609, AK068616, AK067910, and AK103553 (Fig. 1a). OsDMA1 paralogs were selected based on (i) sequence similarity (AK102609), (ii) up-regulation by Fe deficiency (AK067910, AK102609, and AK103553), or (iii) presence of LWDGEI at the C terminus (AK068616). OsDMAS1, TaDMAS1, and HvDMAS1 all possess LWDGEI at their C terminus (see Supplementary Fig. S1 at *JXB* online) while in ZmDMAS1 aspartic acid is replaced by alanine (Bashir *et al.*, 2006). AK102609 showed 65% homology to OsDMAS1, while AK068616 showed 46% homology. AK067910 and AK103553 both showed 24% homology to OsDMAS1 based on amino acid sequence. We previously reported that AK102609 does not have DMAS activity (Bashir *et al.*, 2006). Among these selected clones, the substrate-binding sites of AK102609 and AK068616 have significant similarity to OsDMAS1 (Fig. 1b, Supplementary Fig. S1). The substrate-binding domain of OsDMAS1 was proposed as AHYWHWVKAMGYS (Bashir *et al.*, 2006), whereas the substrate-binding sites of AK102609 and AK068616 were proposed as AAYWHWVDFMGYS and AIYWHWVGALGYS, respectively (Fig. 1b). The expression of *AK103553* is up-regulated by Fe deficiency in both roots and shoots, as revealed by a microarray analysis, while the expression of *AK067910* and *AK102609* is specifically up-regulated in roots and shoots, respectively (Bashir *et al.*, 2014). OsDMAS1 and HvDMAS1 displayed *in vitro* DMAS activity in line with our previous report (Bashir *et al.*, 2006), whereas AK102609, AK067910, AK068616, and AK103553 did not show DMAS activity (Fig. 1c). These results suggest that rice may only have one functional DMAS.

### Characterization of the *OsDMAS1* knock-down mutants

**Fig. 2.**
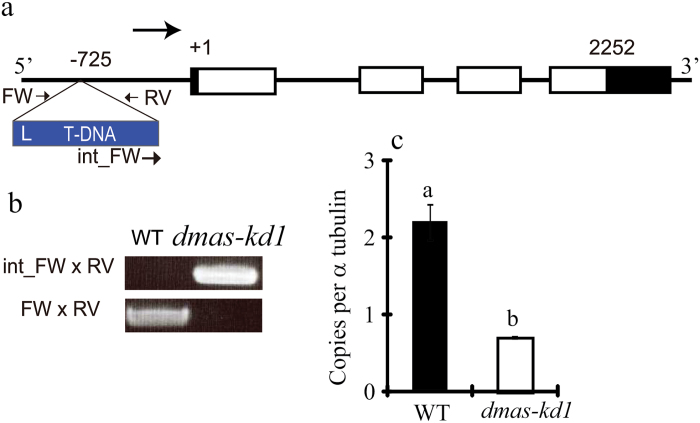
Characterization of *OsDMAS1* knock-down (*dmas-kd1*) plants. (a) Integration of T-DNA in the promoter of *OsDMAS1*. White boxes represent exons, while black boxes show 3′ and 5′ untranslated regions. (b) Confirmation of integration of T-DNA and the homozygous status of *dmas-kd1*; primer positions are shown in (a). (c) Expression of *OsDMAS1* in WT and *dmas-kd1*. Different letters indicate statistically different values according to a Student–Newman–Keuls (SNK) test (*P*<0.05; *n*=4).

To characterize the role of *OsDMAS1* in the Fe-deficiency response, T-DNA mutants of *OsDMAS1* were obtained and T-DNA integration was confirmed by PCR using internal primers for T-DNA and *OsDMAS1*, or primers surrounding the T-DNA integration site (Fig. 2a, b; Supplementary Figs S2a, b and S3a, b). We first characterized the *dmas-1* mutant in which T-DNA was integrated into the second intron of *OsDMAS1* (see Supplementary Fig. S2); however, we could not find any homozygous plants for this mutant after analysing DNA from 19 independent plants harboring T-DNA in their genome (data not shown), indicating that knock-out mutants of *OsDMAS1* may be lethal. Segregation analyses were also performed on seeds harvested from *dmas-1* heterozygous plants. These analyses revealed the presence of 3.7% homozygous seeds in contrast to expected proportion of 25% (Supplementary Fig S2c). Our data also suggested that the *dmas-1* mutant line contained more than one copy of T-DNA, so further experimentation involving independent knock-out lines is needed to confirm the lethal phenotype of the *dmas-ko* mutant. It should be noted that according to the Rice Annotation Project database (http://rapdb.dna.affrc.go.jp/), the other copy of T-DNA is not integrated into any gene. As we failed to find homozygous mutants for DMAS knock-out plants, we instead characterized DMAS knock-down plants. In *dmas-kd1*, T-DNA was integrated ~725 bp upstream of the start codon (Fig. 2a). The integration of T-DNA as well as the homozygous status of *dmas-kd1* was confirmed using genomic PCR (Fig. 2b). Real-time PCR analysis revealed that *OsDMAS1* expression was significantly down-regulated in the *dmas-kd1* mutant (Fig. 2c). In the *dmas-kd2* mutant, T-DNA was ~252 bp upstream of the start codon (see Supplementary Fig. S3). The integration of T-DNA and the homozygous status of *dmas-kd2* was confirmed by genomic PCR (Supplementary Fig. S3b). The expression of *OsDMAS1* was significantly reduced in *dmas-kd2* compared to WT plants (Fig. S3c). Root and shoot length was significantly reduced in *dmas-kd2* plants under both Fe-sufficient and Fe-deficient conditions (Fig. S3d–h). Seed yield was also significantly reduced in *dmas-kd2* compared to WT plants (Fig. S3j). In addition to the low yield, we also found germination problems with *dmas-kd2*; consequently, *dmas-kd1* was selected for detailed molecular and morphological analysis.

**Fig. 3.**
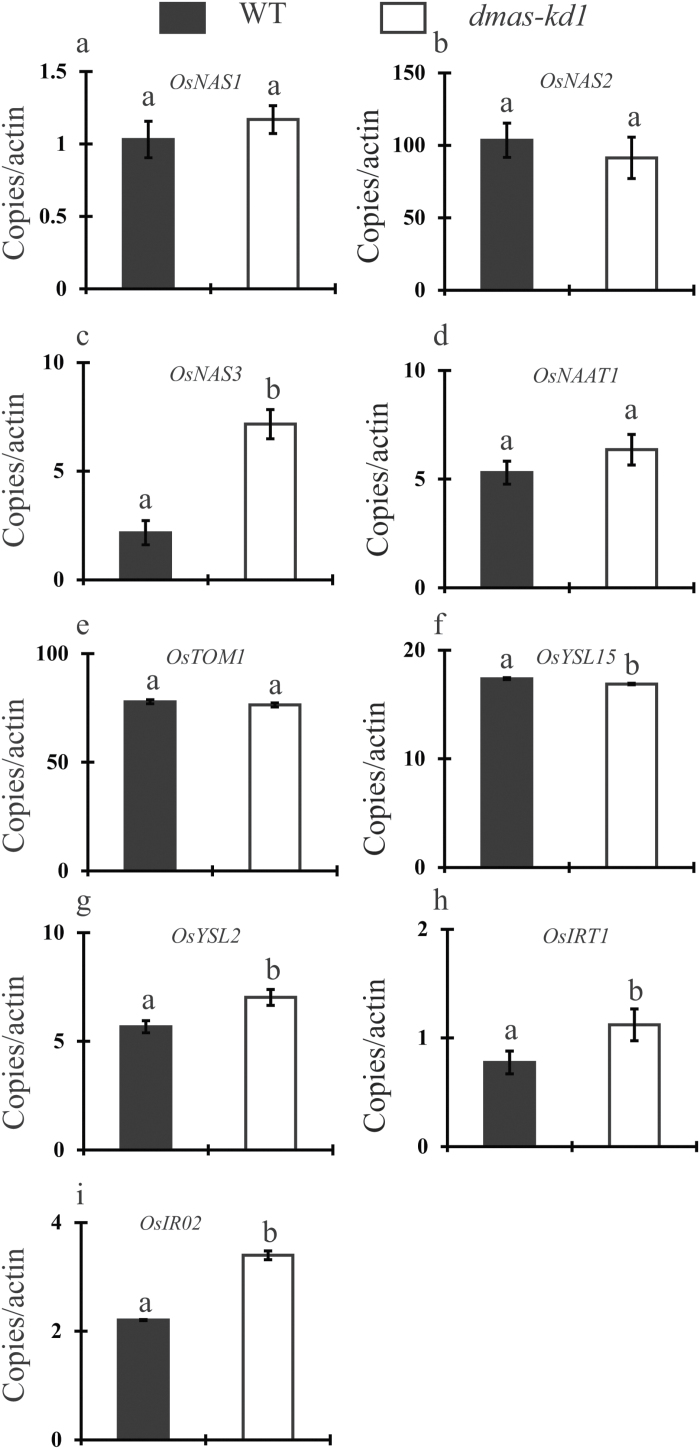
Changes in the expression of Fe homeostasis-related genes in WT and *dmas-kd1* plants. Changes in the relative expression of (a) *OsNAS1*, (b) *OsNAS2*, (c) *OsNAS3*, (d) *OsNAAT1*, (e) *OsTOM1*, (f) *OsYSL15*, (g) *OsYSL2*, (h) *OsIRT1*, and (i) *OsIRO2* in Fe-deficient roots of *dmas-kd1* compared to the WT. The values are normalized to the expression of *OsActin1*. Different letters indicate values that are statistically different from each other according to a Student–Newman–Keuls (SNK) test (*P*<0.05; *n*=3).

**Fig. 4.**
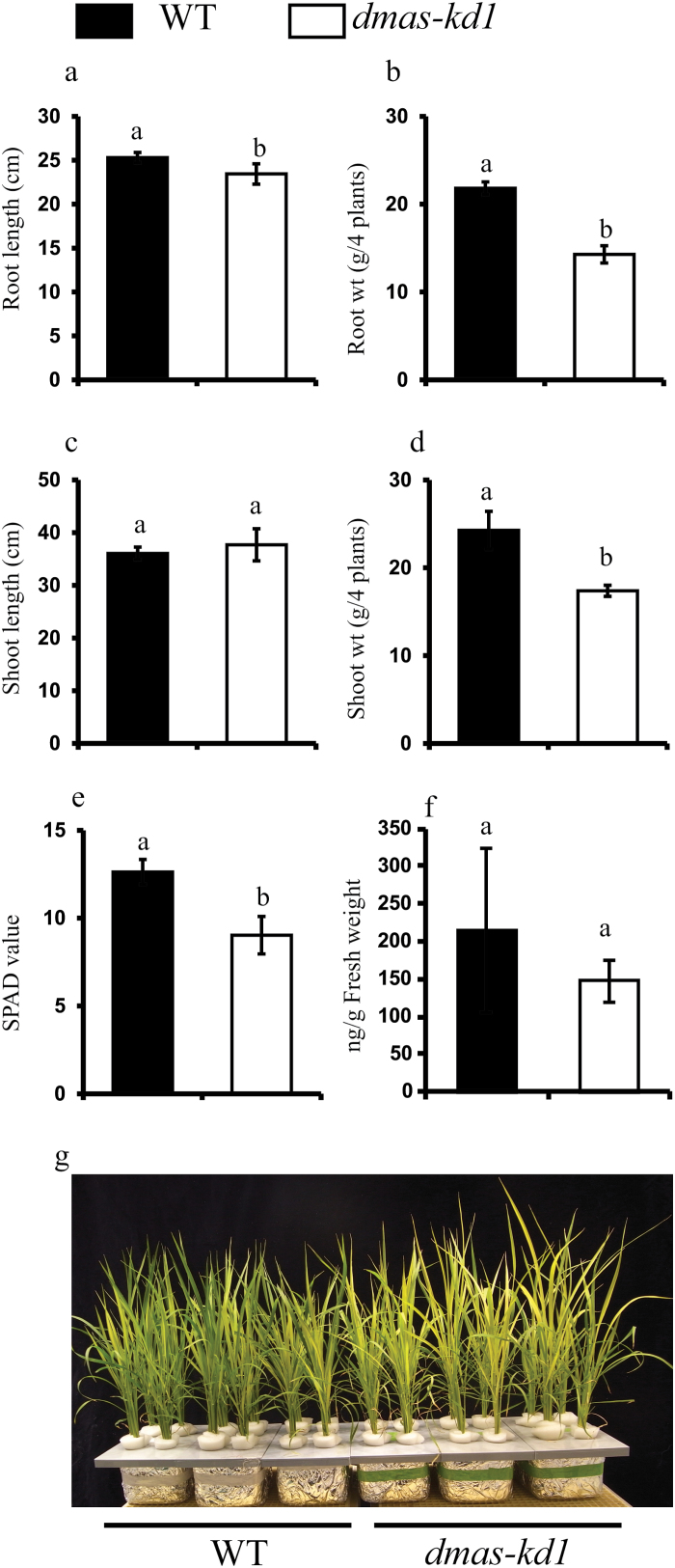
Morphological characteristics of WT and *dmas-kd1* plants grown under Fe-deficient conditions. (a) Root length, (b) root fresh weight, (c) shoot length, (d) shoot fresh weight, (e) chlorophyll content (SPAD meter value), and (f) DMA secretion from roots of WT and *dmas-kd1* plants. Different letters indicate values that are statistically different from each other according to a Student–Newman–Keuls (SNK) test (*P*<0.05; *n*=4, except f where *n*=6). (g) Phenotype of hydroponically grown 4-week-old WT and *dmas-kd1* plants.

Similar to *dmas-kd2*, the germination rate of *dmas-kd1* was also significantly reduced compared to WT plants. The germination rate of *dmas-kd1* was recorded as 21.2 ± 8.7% compared to 95.8 ± 5.5% in WT plants. The expression pattern of selected Fe homeostasis-related genes was characterized in the *dmas-kd1* mutant. No difference was observed for *OsNAS1*, *OsNAS2*, *OsNAAT1*, and *TOM1*. The expression of *OsNAS3*, *OsYSL2*, *OsIRT1*, and *OsIRO2* was significantly up-regulated in the *dmas-kd1* mutant (Fig. 3), suggesting that metal homeostasis is significantly disturbed in this mutant. The shoot length of *dmas-kd1* was similar to WT plants, whereas the root length as well as root and shoot fresh weight of *dmas-kd1* plants was significantly reduced compared to the WT grown under Fe-deficient conditions (Fig. 4a–d). The chlorophyll content was similar in WT and *dmas-kd1* plants when grown under Fe-sufficient conditions (data not shown); however, *dmas-kd1* plants were more chlorotic compared to the WT when grown under Fe-deficient conditions (Fig. 4e, g). The secretion of DMA from the roots of *dmas-kd1* was slightly less compared to WT plants; however, the results were not statistically significant (Fig. 4f, Supplementary Fig. S4). Similarly, we failed to find statistically significant differences for endogenous DMA concentrations (data not shown).

### Metal profiling of *dmas-kd1* plants

**Fig. 5.**
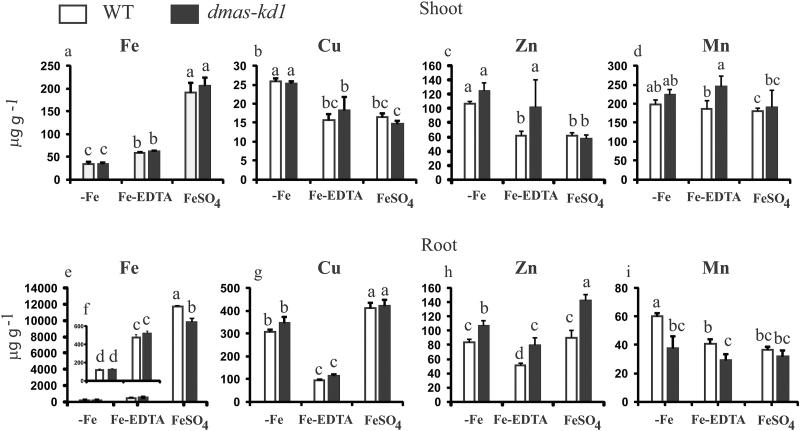
Metal concentrations of WT and *dmas-kd1* plants grown under Fe-deficient (–Fe), Fe-EDTA (100 mM Fe-EDTA), or FeSO_4_ (10 μM FeSO_4_) conditions. (a–d) Shoots: (a) Fe, (b) Cu, (c) Zn, and (d) Mn. (e–i) Roots: (e, f Fe, (g) Cu, (h) Zn, and (i) Mn. For Fe, (f) is an enlarged view of the first two treatments in (e). Different letters indicate values that are statistically different from each other according to a Student–Newman–Keuls (SNK) test (*P*<0.05; *n*=4).

WT and *dmas-kd1* plants were grown either without Fe or with 100 µM Fe-EDTA or 10 µM FeSO_4_. While no difference was observed for shoot Fe accumulation between WT and *dmas-kd1* plants, significant differences were observed between plants cultured in the presence of 10 µM FeSO_4_ and those grown in the presence of 100 µM Fe-EDTA (Fig. 5a). WT plants supplied with 10 µM FeSO_4_ accumulated 3.3 times more Fe in their shoots compared to plants grown in the presence of 100 µM Fe-EDTA. The *dmas-kd1* plants accumulated less copper (Cu) in their shoots when grown in the presence of 10 µM FeSO_4_ than the *dmas-kd1* plants supplied with Fe-EDTA, whereas no difference was observed in Cu accumulation between WT and *dmas-kd1* plants (Fig. 5b). Surprisingly, *dmas-kd1* plants accumulated more Zn and manganese (Mn) in their shoots than WT plants when Fe was supplied in the form of Fe-EDTA (Fig 5c, d). In roots, *dmas-kd1* plants accumulated less Fe than WT plants when supplied with FeSO_4_. WT plants grown in the presence of FeSO_4_ accumulated 25 times more Fe in their roots than plants supplied with Fe-EDTA, whereas this ratio was 19 for *dmas-kd1* plants (Fig. 5e, f). No significant difference was observed in Cu accumulation between WT and *dmas-kd1* plants; however, plants supplied with 10 μM FeSO_4_ accumulated significantly more Cu in roots compared to plants supplied with Fe-EDTA (Fig. 5g). Significant differences in Zn accumulation were observed between WT and *dmas-kd1* plants grown under all treatments. The *dmas-kd1* plants accumulated more Zn in their roots than the WT plants under Fe-deficient, Fe-EDTA, and FeSO_4_ conditions (Fig. 5h). The *dmas-kd1* plants accumulated less Mn in their roots than WT plants when grown under Fe-deficient conditions or when supplied with 100 µM Fe-EDTA (Fig. 5i).

**Fig. 6.**
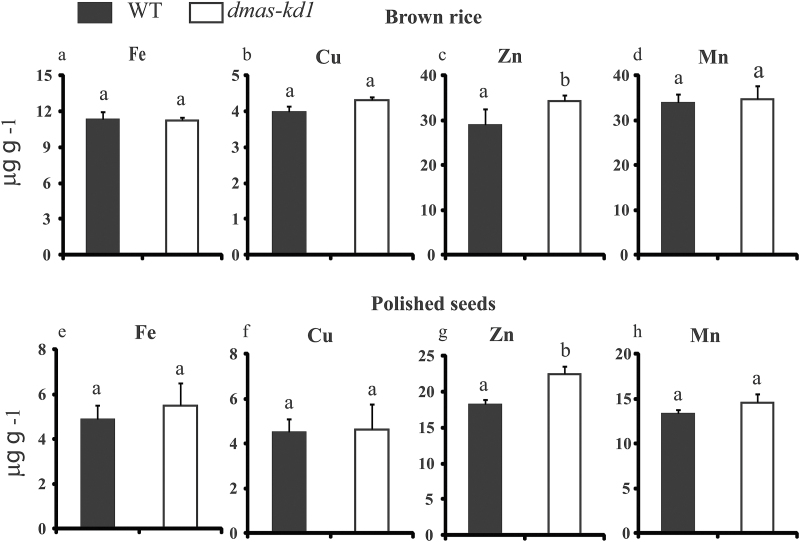
Seed metal concentrations of WT and *dmas-kd1* plants for (a–d) brown rice (dehusked rice seeds) and (e–h) polished rice seeds. (a, e) Fe, (b, f) Cu, (c, g) Zn, and (d, h) Mn. Different letters indicate values that are statistically different from each other according to a Student–Newman–Keuls (SNK) test (*P*<0.05; *n*=4).

We also investigated whether metal concentrations in seeds of *dmas-kd1* plants significantly differed from those of the WT, and for this purpose concentrations were also measured in soil-grown WT and *dmas-kd1* plants (Fig. 6). In dehusked rice seeds (brown rice), no differences were observed in Fe, Cu, or Mn accumulation; however, *dmas-kd1* seeds accumulated significantly higher Zn levels (Fig. 6a–d). A similar trend was also observed for white rice (polished seeds), where the Zn concentration was significantly higher in *dmas-kd1* compared to WT seeds, whereas no differences were observed for Fe, Cu, or Mn accumulation (Fig. 6e–h).

## Discussion

The mechanisms of metal uptake from the rhizosphere and transport to aerial parts and then to the developing panicles/seeds is reasonably well understood in rice (Bashir *et al.*, 2013*a*, 2013*b*; Kobayashi *et al.*, 2014). Rice is reported to utilize a partial Strategy I as well as Strategy II for Fe uptake and transport within the plant body (Ishimaru *et al.*, 2006; Bashir *et al.*, 2010). The components of Fe uptake and translocation work in strong coordination in response to Fe-deficiency (Kobayashi *et al.*, 2014). Thus, if one transporter/transport system is disturbed, other systems are up-regulated to complement Fe transport (Ishimaru *et al.*, 2012*b*). Moreover, these transporters often transport more than one metal, for example OsIRT1 and OsNRAMP1 transport Fe and Cd (Ishimaru *et al.*, 2006; Nakanishi *et al.*, 2006; Takahashi *et al.*, 2011), whilst OsNRAMP5 also transports Fe in addition to Mn and Cd (Ishimaru *et al.*, 2012*a*, 2012*b*; Sasaki *et al.*, 2012). Thus, the knock-down/knock-out of one transporter/transport system may be complemented by others, but it may also affect the distribution of other metals (Ishimaru *et al.*, 2010, 2012*a*, 2012*b*).

OsDMAS1 is a member of the aldo-keto reductase (AKR) subfamily 4B (AKR4B8). AKRs have been reported in mammals, amphibians, plants, yeast, protozoa, and bacteria, and they catalyse a diverse range of substrates, including aliphatic and aromatic aldehydes, monosaccharides, steroids, prostaglandins, and polycyclic aromatic hydrocarbons (Jez *et al.*, 1997). AKRs are monomeric proteins that are around 320 amino acids long, and they utilize NADPH as a cofactor (Jez *et al.*, 1997). AKRs have conserved NADPH-binding domains, even in proteins sharing 30% homology (Jez *et al.*, 1997). The variation in substrate-binding domain is critical for selecting the right substrate, and any mutation in the domain may lead to non-specific activity (Jez *et al.*, 1997). Because members of the AKR family are functionally redundant, there is a chance that other members may display DMAS activity. The existence of multiple genes for *NAS* and *NAAT* in graminaceous crops also supports this hypothesis (Inoue *et al.*, 2003, 2008). We analysed four paralogs of OsDMAS1 for DMA synthesis activity, one of which (AK102609) was already described as lacking DMAS activity (Bashir *et al.*, 2006). *OsDMAS1* encodes a protein of 318 amino acids, whereas *AK102609*, *AK068616*, *AK103553*, and *AK067910* putatively encode polypeptides of 321, 325, 355, and 377 amino acids, respectively. The similarity among graminaceous DMASs ranges from 82–97.5%, while the rice paralogs used in the current studies exhibited 24–65% homology to OsDMAS1.

The various microarray analyses performed in our laboratory have shown that the expression of *AK103553* is up-regulated by Fe deficiency in both roots and shoots (Bashir *et al.*, 2014), whereas the expression of *AK102609* and *AK067910* is induced by Fe deficiency specifically in leaves and roots, respectively (data not shown). We previously reported sequence variation among graminaceous DMASs, whereas the substrate-binding domain is strictly conserved (Bashir *et al.*, 2006; Bashir and Nishizawa, 2006). Because ZmDMAS1 has threonine at position 127 (OsDMAS1) instead of lysine and still displays enzyme activity (see Supplementary Fig. S1), this site is not conserved for DMAS activity. There were two more substitutions on the substrate-binding site in AK068616 compared to OsDMAS1, namely histidine at position 52 (OsDMAS1) was replaced with isoleucine, and methionine at 226 was replaced with leucine. It has been suggested that the amino acid at position 226 does not play a role in substrate selectivity (Jez *et al.*, 1997). Moreover, in AK102609, histidine 52 was replaced with alanine, whereas alanine at positon 128 was replaced with phenylalanine. This suggests that histidine 52 may be very important for DMAS activity. These results also indicate that in rice there seems to be only one functional DMAS. Although there are six genes for NAAT in rice, it has been reported that it mainly depends on OsNAAT1 for the conversion of NA to 3’’-keto acid (Cheng *et al.*, 2007). This is in contrast with barley, which has two functional NAAT genes (Takahashi *et al.*, 1999). Thus, the low production of DMA and the sensitivity to Fe deficiency in rice compared to other graminaceous crops could be due to the presence of only one functional NAAT and DMAS gene (Mori *et al.*, 1991; Bashir *et al.*, 2006; Cheng *et al.*, 2007).

DMA has not only been detected in root exudates, but also in roots, shoots, and seeds, as well as in the xylem and phloem of rice, and is believed to play a very significant role in metal transport (Masuda *et al.*, 2009; Nozoye *et al.*, 2011; Nishiyama *et al.*, 2012). DMAS knock-down plants secreted 29% less DMA from their roots compared to WT plants; however, these results were not statistically significant (Fig. 4f). Similarly, we could not detect statistically significant differences for endogenous DMA in roots compared to WT plants. It should be noted that DMAS knock-out plants seemed to result in a lethal phenotype, and we had to choose knock-down plants exhibiting mild phenotypes. Rice plants overexpressing barley *HvNAAT* genes produce/secrete significantly higher amounts of DMA and are tolerant to low Fe availability under alkaline conditions (Takahashi *et al.*, 2001). Mutations in *OsNAAT1* significantly disturb DMA synthesis/secretion and result in Fe-related phenotypes (Cheng *et al.*, 2007); however, mutations in *OsDMAS1* genes have not been characterized. Our results indicate that rice seems to harbors only one functional gene encoding DMA. Changes in the metal profiling and expression of metal homeostasis-related genes highlight the role of *OsDMAS1* in metal homeostasis in rice. It should be noted that although knock-out of *OsNAAT1* does not produce DMA, it is significantly different from *OsDMAS1* knock-out. This difference can be explained by the fact that the knock-out of *OsNAAT1* fails to convert nicotianamine (NA) to 3’’-keto acid and, as a result, the plants could accumulate more NA than the WT; thus, deficiency of DMA may be complemented by an increased accumulation of NA. NA is a structural analog of MAs, and is responsible for metal homeostasis through metal translocation in both graminaceous plants (Kakei *et al.*, 2009; Nishiyama *et al.*, 2012) and dicot plants (Takahashi *et al.*, 2003; Schuler *et al.*, 2012). In *DMAS* knock-out mutants, NA is converted into 3’’-keto acid, which may have no apparent physiological role in plants; however, this 3’’-keto acid cannot be converted into DMA. Thus, theoretically, *OsDMAS1* knock-out plants are at more of a disadvantage than *OsNAAT1* knock-out plants. This might explain why we could not find any homozygous knock-out plants (see Supplementary Fig. S2), leading us to characterize *OsDMAS1* knock-down plants instead.

The expression of *OsDMAS1* was significantly reduced in *dmas-kd1* (Fig. 2c). In *dmas-kd1* grown under Fe-deficient conditions, the expression of *OsNAS3*, *OsYSL2*, *OsIRT1*, and *OsIRO2* was significantly up-regulated compared to WT plants (Fig. 3). OsNAS3 is a functional NA synthase, and its expression significantly differs from that of *OsNAS1* and *OsNAS2* in both roots and shoots. In roots, the expression of *OsNAS3* is up-regulated by Fe deficiency; however, it is very low compared to that of *OsNAS1* and *OsNAS2*. In shoot tissue, where *OsNAS1* and *OsNAS2* are up-regulated by Fe deficiency, the expression of *OsNAS3* is suppressed (Inoue *et al.*, 2003). OsIRT1 contributes significantly to Fe uptake in rice. The up-regulation of *OsIRT1* indicates that the phenotype of *dmas-kd1*, which may result in reduced Fe(III)-DMA uptake, could be effectively complemented by Fe^2+^ uptake. Similarly, the up-regulation of *OsYSL2*, which transports Fe(II)-NA and Mn(II)-NA, and is important for the phloem translocation of Fe and Mn (Koike *et al.*, 2004; Ishimaru *et al.*, 2010), also indicates that Fe-NA transport within the body of the plant may increase to complement the decrease in transport of Fe(III)-DMA.

Because contrasting results have been reported for the determination of metal transport-related genes while using different sources of Fe, such as Fe-EDTA versus FeSO_4_ (Ishimaru *et al.*, 2012*b*; Sasaki *et al.*, 2012; Takahashi *et al.*, 2014), we used both these Fe sources to examine the differences in metal accumulation. The expression of genes is regulated distinctly for Fe-EDTA versus FeSO_4_ (Takahashi *et al.*, 2014), and hence in this study we used these Fe sources to compare the metal accumulation and to characterize the response of WT and knock-down plants. Our results clearly indicate that metal homeostasis is significantly disturbed in *dmas-kd1* compared to WT plants. This is despite the fact that only the Mn concentration was significantly less in *dmas-kd1* plants, whereas the root Zn concentration was significantly higher compared to WT plants, and no apparent difference was observed in Fe or Cu concentrations. The Zn concentration significantly increased not only in roots and shoots, but also in brown seed and polished seed. This could be linked to the up-regulation of *OsNAS3*, which would lead to the increased synthesis of NA. Although no difference was observed in the Fe concentration between WT and *dmas-kd1* plants, the chlorotic phenotype and up-regulation of Fe-deficiency genes indicates that Fe may only be partially available for physiological functions under Fe-limited conditions, and *dmas-kd1* plants could effectively sense the availability of Fe and regulate Fe deficiency-related genes (Fig. 3).

Important differences were also observed between WT plants supplied with either Fe-EDTA or FeSO_4_ (Fig. 5). Plants supplied with FeSO_4_ accumulated much more Fe in their shoots than those supplied with Fe-EDTA. This Fe accumulation was even greater than that recorded in plants grown under excess Fe conditions (500 μM Fe-EDTA) (Bashir *et al.*, 2014). Moreover, this high accumulation of Fe did not disturb Cu or Zn accumulation in the shoot tissue, and only the Mn concentration was slightly reduced in plants supplied with 10 μM FeSO_4_ compared to those supplied with 100 μM Fe-EDTA. In root tissue, *dmas-kd1* plants accumulated significantly less Fe when supplied with FeSO_4_, and accumulated significantly more Zn compared to WT plants, when grown under Fe-deficient or Fe-sufficient conditions (Fig. 5h. These results clearly suggest that it is not reasonable to compare the phenotype of mutants when using different sources of Fe.

It should be noted that *dmas-kd1* are knock-down plants, and we failed to find a homozygous line when T-DNA was integrated into the second intron of *OsDMAS1*. Thus, reduction in the expression of *OsDMAS1* could be complemented by other genes involved in the Fe^2+^ or NA metal complex. The increased Zn concentration in shoots and seeds could be attributed to the increased expression of *OsNAS3*. It has already been reported that increased expression of *OsNAS3* increases seed Zn and Fe concentrations (Lee *et al.*, 2009*b*; Johnson *et al.*, 2011). In the phloem, Fe is predominantly found as Fe-DMA, whereas Zn is found as Zn-NA (Nishiyama *et al.*, 2012). Although there was no difference in seed Fe concentration between *dmas-kd1* and WT plants following Fe accumulation, the increased expression of *OsNAS3* may have complemented the possible decrease in Fe accumulation in *dmas-kd1* due to the decreased expression of DMAS. Our results clearly indicate that *OsDMAS1* is very important for Fe homeostasis, and that decrease in expression of *OsDMAS1* significantly disturbs the expression of genes involved in Fe uptake and homeostasis.

## Supplementary data

Supplementary data are available at *JXB* online.

Fig. S1. Sequence homology among graminaceous *DMAS*.

Fig. S2. Characterization of *OsDMAS1* knock-out plants (*dmas-1*) plants.

Fig. S3. Characterization of *OsDMAS1* knock-down 2 (*dmas-kd2*) plants.

Fig. S4. DMA secretion from WT and *dmas-kd1* plants grown under Fe-deficient conditions.

## Supplementary Material

Supplementary_Figures_S1_S4
