# Supplementary material for: Paralogs and mutants show that one DMA synthase functions in iron homeostasis in rice
Source: J Exp Bot. 2017 Mar 28;68(7):1785–95. doi: 10.1093/jxb/erx065 (PMC5444454; doi:10.1093/jxb/erx065)
Supplement: Supplementary_Figures_S1_S4 [file erx065_suppl_Supplementary_Figures_S1_S4.pdf]

Fig.-S1. Sequence Homology Among Graminaceous *DMAS*

```
OsDMAS1  MSDGGAGAKG AGFGMPRVGM GTAVQGPRPE PIRRAVLKAI EAGYRHFDTA AHYETEAPIG EAAAEAVRSG
HvDMAS1  M---GAGDRT VA-GMPRIGM GTAVQGPKPD PIRRAVLRAI EIGYRHFDTA AHYETEAPIG EAAAEAVRSG
TaDMAS1  M---GAGDKT AA-GMPRIGM GTAVQGPKAD PIRRAVLRAI QVGYRHFDTA AHYETEAPIG EAAAEAVRSG
ZmDMAS1  M---SATGRA PC-GLPRVGL GTAVQGPRPD PVRAAVLRAI QLGYRHFDTA AHYATEAPIG EAAAEAVRTG
AK068616 M--ARCFVLN TGAKIPSVGL GTWQAEPGVV --GNAVYAAV KAGYRHIDCA QAYFNEKEIG VALKKVFDEG

OsDMAS1  AIASRADPFI TSKLWCSDAH RDRVLPALRQ TLWNLQMEYV DLYLVHWPVS MKPGRYKAPF TADDFVPFDM
HvDMAS1  AVASRDDLFI TSKLWCSDAH GDRVVPALRH TLRNLQMEYV DLYLVHWPVS MKPGRFKAPF TAEDFVPFDM
TaDMAS1  AVASRDELFI TSKLWCSDAH RDRVVPALRQ TLRNLQMEYV DLYLVHWPVS MKPGRFKAPF TADDFVPFDM
ZmDMAS1  LVASREDLFV TSKVWCADAH RDRVLPALRR TLSNLQMEYV DLYMVHWPVT MKAGRFTAPF TPEDFEPFDM
AK068616 IV-KREDIFI TSKLWCTNHA PEDVPVALDS TLQDLQTDYV DLYLIHWPVR MK---KGAGF GGQNVLPTDI

OsDMAS1  RAVWEAMEEC HRLGLAKAIG VCNFSCKKLD TLLSFATIPP AVNQVEVNPV WQQRKLRELC REKGVQICAY
HvDMAS1  RAVWEAMEEC HRLGLAKAIG VANFSCKKLD TLLSFATIPP AVNQVEVNPV WQQRKLREFC RGKGIQLCAY
TaDMAS1  RAVWEAMEEC HRLGLAKAIG VANFSCKKLE TLLSFATIPP TVNQVEVNPV WQQRKLREFC RGKGIQLCAY
ZmDMAS1  RAVWEAMEEC HRLGLAKAIG VCNFSCKKLE TLLSFATIPP VVNQVEINPV WQQRKLREFC RAKGIQLCAY
AK068616 PATWAAMEKL HDSGKARAIG VSNFSSKKLE DLLAVARVPP AVDQVECHPV WQQTKLRKFC TSKGIHLSAY

OsDMAS1  SPLGASGTHW GSDSVMASAV LRDIAQSKGK TVAQACLRWV YE-QGDCLIV -KSFDEARMR ENLDIVGWE
HvDMAS1  SPLGAKGTHW GSDAVMDAGV LQDIAASRGK SVAQVCLRWV YE-QGDCLIV -KSFDEARMR ENLDVDGWE
TaDMAS1  SPLGAKGTHR GSDAVMDAGV LQEIAASRGK SVAQVCLRWV YE-QGDCLIV -KSFDEARMR ENLEVDGWE
ZmDMAS1  SPLGAKGTHW GSDSVMDSGV LHEIAKSKGK TVAQVCLRWV YE-QGDCLIV -KSFDEGRMK ENLDIVDWE
AK068616 SPLGSPGTAS VKAVGNVLAH PVVVSTAEKL GKTPAQVALR WGIQMGHSVL PKSTHEERIK ENIDVYDWS

OsDMAS1  LTEEERQRIA GIPQRKINRA LRFVSDHGP YKSLDDLWDGE I*
HvDMAS1  LTEEERRRIA EIPQRKINLG KRYVSDHGP YKSLEELWDGE I*
TaDMAS1  LTEEERLRIA EIPQRKINLG KRYVSEHGP YKSLEELWDGE I*
ZmDMAS1  LSEEERQRIS KIPQRKINQG RRYVSEHGP YKSFEELWAGE I*
AK068616 IPEDLFIKLS EIEQMKLIRG EFWTHPEGV YKSIEELWDGE I*
```

NADPH binding Domain

Substrate Binding Domain

Conserved sequence

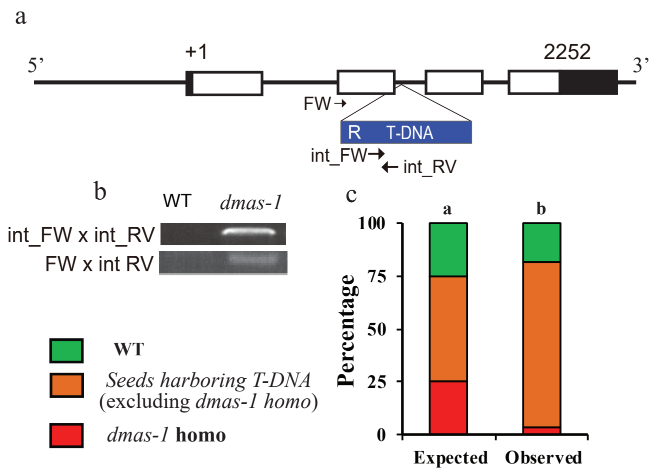

Fig.-S2. Characterization of *OsDMAS1* knock out (*dmas-1*) plants

a). Integration of T-DNA in 2nd intron of *OsDMAS1*, white boxes reresent exons, while black boxes show 3' and 5' untranslated region. The direction of transcription is shown by arrow. b). Confirmation of integration of T-DNA in *dmas-1*, primer positions are shown in a. b). Confirmation of integration of T-DNA in *dmas-1*, primer positions are shown in a. c). Segregation analysis of *dmas-1* heterozygous plants using *dmas-1* seeds (n=24). The observed ratio is significantly different from expected ratio according to chi square test ($p<0.01$).

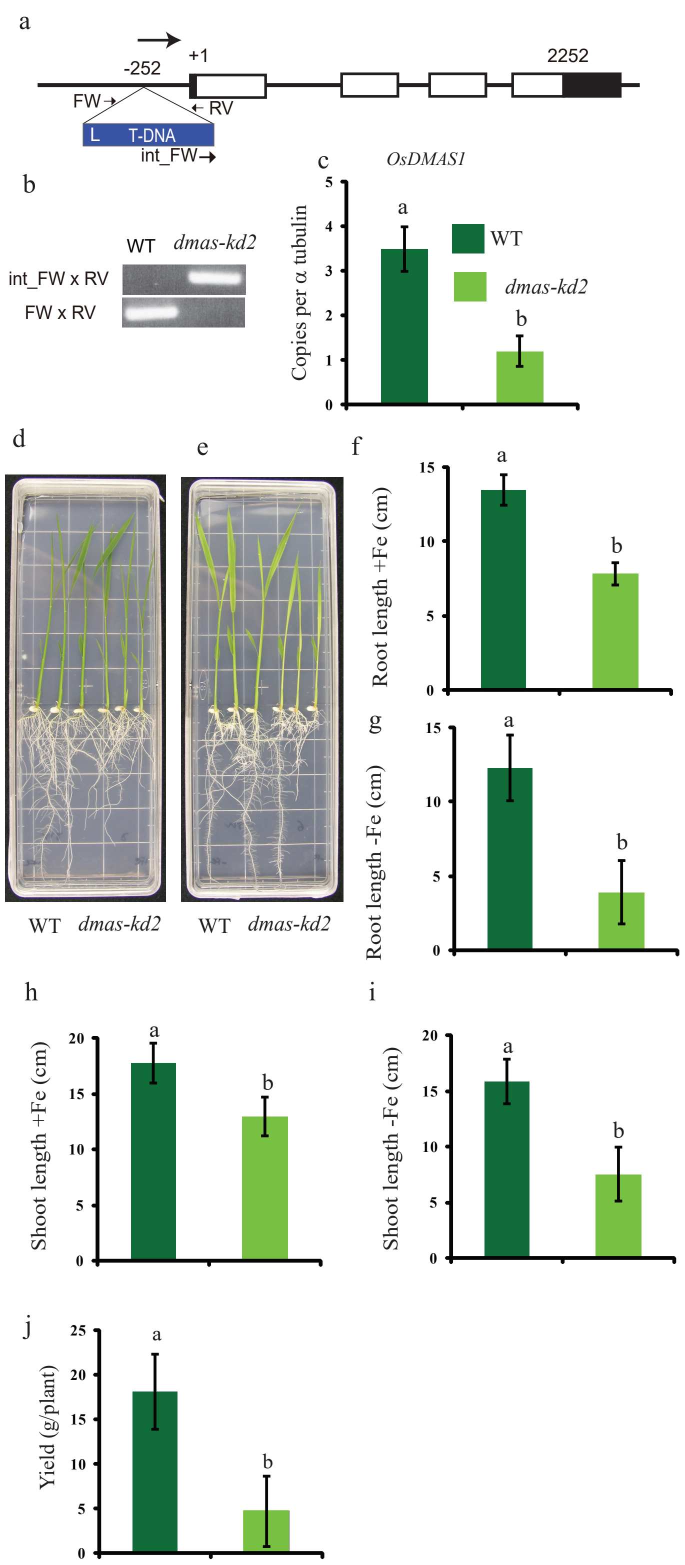

Fig.-S3. Characterization of *OsDMAS1* knock down (*dmas-kd2*) plants

a). Integration of T-DNA in promoter of *OsDMAS1*, white boxes resersent exons, while black boxes show 3' and 5' untranslated region. The direction of transcription is shown by arrow. b). Confirmation of integration of T-DNA and homozygous status of *dmas-kd2*, primer positions are shown in a. c). Expression of *OsDMAS1* in WT and *dmas-kd2.* Phenotype of WT and *dmas-kd2* plants grown in the presecen (d) and absecne (e) of Fe. Root length (cm) of WT and *dmas-kd2* plants grown in the presence (f) and absence (g) of Fe. Shoot length (cm) of WT and *dmas-kd2* plants grown in the presence (h) and absence (i) of Fe. J). Yield of soil grown plants of WT and *dmas-kd2*. Column bars followed by different letters are statistically different from eachother according to SNK test ($p<0.05$; $n=4$)

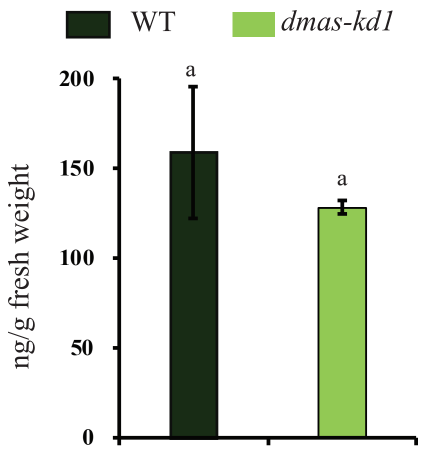

Fig.-S4. DMA secretion from WT and *dmas-kd1* plants grown under Fe deficient conditions.
DMA secretion from roots of WT and dmas-kd1 plants in an independent experiment. The experimental conditions were same as shown in Fig. 4f.
